# Assessment of visual morbidity amongst diabetic retinopathy at tertiary eye care center, Nepal: a cross-sectional descriptive study

**DOI:** 10.1186/s12886-017-0656-3

**Published:** 2017-12-28

**Authors:** Araniko Pandey, Gyanendra Lamichhane, Roshija Khanal, Salma K. C. Rai, Arjun Malla Bhari, Davide Borroni, Narayan Gautam

**Affiliations:** 1 Lumbini Eye Institute, Bhairahawa, Nepal; 2 Riga Stradins University, Riga, Latvia; 3 Universal College of Medical Sciences, Bhairahawa, Nepal

**Keywords:** Diabetes mellitus, Diabetic retinopathy, Preventable blindness

## Abstract

**Background:**

Diabetic retinopathy (DR) is one of the leading causes of preventable blindness in low and middle income countries. In Nepal, there are less studies regarding DR and they too are limited around Kathmandu valley. This study was done to assess visual morbidity in patients with DR at a peripheral tertiary eye care center of Nepal.

**Methods:**

This was a prospective, hospital based, cross-sectional study in which all consecutive cases of DR were evaluated. DR was classified according to Early Treatment Diabetic Retinopathy Study Research Group - *report no. 10 Table A5–1* (Modified Airlie House Classification). Data entry and analysis was done in an SPSS unit version 20. Wherever applicable, variables were set as 100 eyes.

**Results:**

Total number of patients included in this study was 50. Commonest age group was 50–69 yrs. (43/77 yrs.; min/max) comprising 80% of the total population (*n* = 50) and the predominant population was male (76%). Non proliferative diabetic retinopathy (NPDR) was found in 69%, proliferative diabetic retinopathy (PDR) in 31% and advanced diabetic eye disease (ADED) in 3% (*n* = 100).

**Conclusions:**

All the stages of DR were present at significant proportions in this study, noteworthy was the percentage of PDR. This study shows an urgency to gather a national data on DR, raise awareness among diabetics and train effective man power at a local level to diagnose DR at an early stage.

## Background

Diabetic retinopathy (DR) is emerging globally as one of the main causes of avoidable blindness and a leading cause of blindness in low and middle income countries. This might be due to industrialization, mobilization of population and changing life styles [1].

Blindness from DR can be prevented by early diagnosis, optimisation of associated risk factors and timely ocular treatment, but systematic screening for DR is rarely done in low and middle income countries (LMIC) like Nepal [2–6].

Various studies have been done abroad regarding DR and its awareness [7–11]. However in Nepal, there are only few studies and they too are limited in and around Kathmandu [3, 12–16]. Despite the studies being conducted in Nepal, data are still not enough to be comparable to the global scenario.

Nepal is a country located in south asia with a population of approx. 25 million [17]. Lumbini Eye Institute (LEI) is situated in the southern belt of Nepal, around 4 km away from the Indian border of Uttar Pradesh. Majority of the patients from Rupandehi and Kapilvastu (approx. 1.4 million population) district are served by LEI. Being a tertiary eye care center, it also serves a fraction of patients who are referred to LEI from further away districts for specialist care. Due to an open border between India and Nepal, a major fraction of patients from the adjoining Uttar Pradesh also visit LEI.

This study was done to enhance the existing scientific knowledge on DR and evaluate its various patterns, at a peripheral tertiary eye care center in Nepal.

## Methods

This was a prospective, hospital based, cross-sectional study done between Aug 2013 to July 2014 at Lumbini Eye Institute. Ethical approval for the study was taken from Institutional Review Board of Lumbini Eye Institute. Verbal and written consent for enrolment was taken from all participants.

Non-probability, convenience sampling method was used to evaluate all consecutive cases of DR. Among them, 50 patients at any stage of DR were selected. Patients with causes other than DR for visual impairment putting diagnosis in dilemma and with poor view of fundus were excluded.

Detailed history taking of all the patients were done. Along with their demographic profile, presence of diminution of vision and duration was recorded. Best corrected visual acuity (BCVA) was recorded using Snellen’s chart. Detailed slit lamp examination of anterior and posterior segment was done. Fundus photography and fundus fluorescein angiography was done, as appropriate. DR was classified according to Early Treatment Diabetic Retinopathy Study Research Group - *report no. 10 Table A5–1* (Modified Airlie House Classification).

Data entry and analysis was done in an SPSS unit version 20. Wherever applicable, variables were set as 100 eyes.

## Results

Commonest age group was 50–69 yrs. (43/77 yrs.; min/max) comprising 80% of the total population (*n* = 50) and the predominant population was male (76%).

Forty two percent (*n* = 100) of the eyes checked had BCVA of <6/60, 48% had visual complaints for >1 yr. Only 4% (2/50) of patients sought for eye check up with no visual complaints (Table 1).

**Table 1:** Patient profile

Categories	Frequency	Percent (%)
Age (yrs) *n = 50*		
40–49	9	18.0
50–59	20	40.0
60–69	20	40.0
70–79	1	2.0
Sex *n = 50*		
Male	38	76.0
Female	12	24.0
Diminution of vision *n = 50*		
Yes	48	96.0
No	2	4.0
Duration of diminution of vision *n = 100*		
<6 months	24	24.0
6 months - <1 yr	20	20.0
>1 yr	48	48.0
No diminution of vision	8	8.0
Best corrected visual acuity *n = 100*		
6/6–6/18	30	30.0
<6/18–6/60	28	28.0
<6/60–3/60	24	24.0
<3/60–1/60	16	16.0
<1/60-PL	2	2.0

*PL* perception of light

**Fig. 1:**
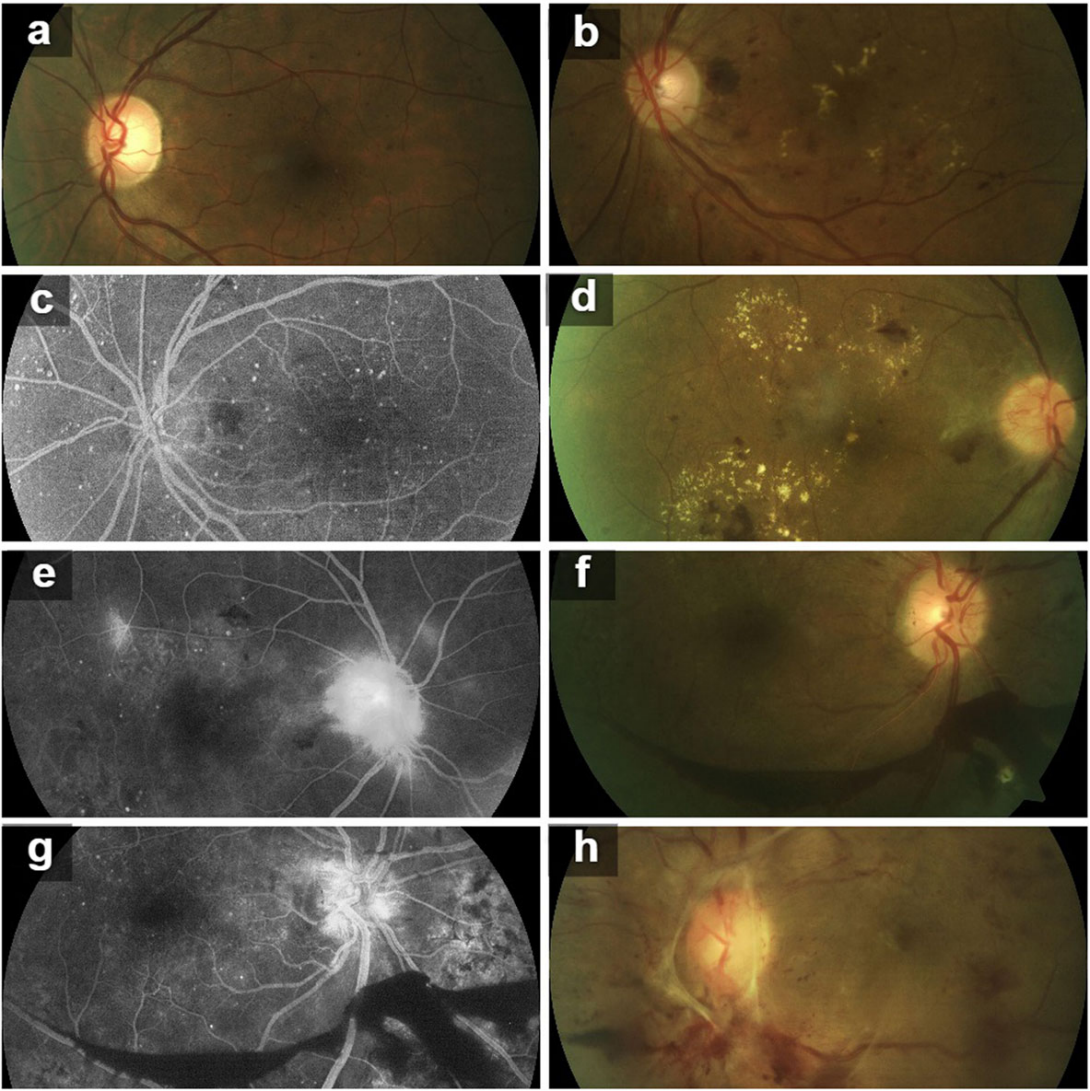
Fundus (**a**, **b**, **d**, **f**, **h**) and fluorescein angiography (**c**, **e**, **g**) photographs. (**a**) Moderate Non Proliferative Diabetic Retinopathy, right eye; (**b**, **c**) Severe Non Proliferative Diabetic Retinopathy, left eye; (**d**, **e**) Early Proliferative Diabetic Retinopathy, right eye; (**f**, **g**) High risk Proliferative diabetic retinopathy, right eye; (**h**) Advanced Diabetic Eye Disease, left eye

Non proliferative diabetic retinopathy (NPDR) was found in 69%, proliferative diabetic retinopathy (PDR) in 31% and advanced diabetic eye disease (ADED) in 3% (*n* = 100). All the stages of DR viz. mild NPDR, moderate NPDR, severe NPDR, early PDR and high risk PDR were seen at 15%, 27%, 27%, 10% and 18%, respectively (*n* = 100) (Table 2 and Fig. 1).

**Table 2:** Distribution of patients with DR

Category of DR (*n = 100*)	Frequency	Percent (%)
NPDR	69	69.0
PDR	31	31.0
Grade of DR		
Mild NPDR	15	15.0
Moderate NPDR	27	27.0
Severe NPDR	27	27.0
Early PDR	10	10.0
High Risk PDR	18	18.0
Advanced Diabetic Eye Disease	3	3.0

*DR* diabetic retinopathy, *NPDR* non proliferative diabetic retinopathy, *PDR* proliferative diabetic retinopathy

## Discussion

Age group and sex predilection in our study was consistent with majority of the studies. This shows that, visual morbidity increases with age and males are more susceptible [15, 16, 18–21].

Majority of the eyes checked (42%, *n* = 100) had decreased BCVA (<6/60) at presentation and with a longstanding visual complaints (>1 yr) in 48% of the study population. These patients presented at a very late stage of DR. Above this, only two patients (*n* = 50) with diabetes sought for an eye check up without any visual complaints. This suggests a lack of awareness regarding visual morbidity related to diabetes. Raising awareness about the consequences of uncontrolled or longstanding diabetes to vision in these patients could change this scenario. Also, counselling and timely referral by their treating physician could play a major role.

There are numerous studies from Nepal to assess the awareness of DR in diabetic population. From the studies on awareness done by Shrestha et al., in 2004; Paudyal G et al., in 2008, Mishra et al., in 2016; to the series of studies by Thapa R et al., from 2012 to 2015; all show decreased awareness [3, 12–14, 22, 23].

Hence, lack of awareness is not only a matter of concern in Nepal and LMIC, but seems to be a global issue, as suggested by various studies from abroad [7, 9, 10, 24–27].

All the stages of DR were present in our study. Percentage of PDR (31%, *n* = 100) and ADED (3%, *n* = 100) is an alarming sign for active intervention that warrants ample community based studies and programs for diagnosis of DR at an early stage. Despite a small sample size, significant proportions of DR at all stages were detected in this study.

To the best of our knowledge, this study has shown the highest percentage of PDR (31%) documented from Nepal. Level of PDR and ADED (34%), as shown in this study, suggests a major backlog of cases at a tertiary as well as primary level. Early diagnosis at the primary level, where ophthalmic assistants may play a key role, and treatment at tertiary center by retina specialists may decrease this burden.

Studies done in Nepal since 2004 till 2016 has shown the prevalence of PDR to be as low as 0.5% to as high as 25.93%. These data however, represent Kathmandu and nearby areas. Studies done outside the valley will certainly contribute to this existing pool of data. The authors would like to encourage researchers from the periphery as well, so that a national data that represents Nepal could be documented [3, 12–16, 23].

## Conclusions

All the stages of DR were present at significant proportions in this study, noteworthy was the percentage of PDR. Attitude towards eye check up in diabetics seemed low. This shows an urgency to gather a national data on DR, raise awareness among diabetics and train effective man power at a local level to diagnose DR at an early stage.

## Abbreviations

ADED: Advanced diabetic eye disease
BCVA: Best corrected visual acuity
DR: Diabetic Retinopathy
NPDR: Non proliferative diabetic retinopathy
PDR: Proliferative diabetic retinopathy
